# Delirium and encephalopathy in severe COVID-19: a cohort analysis of ICU patients

**DOI:** 10.1186/s13054-020-03200-1

**Published:** 2020-08-08

**Authors:** Julie Helms, Stéphane Kremer, Hamid Merdji, Malika Schenck, François Severac, Raphaël Clere-Jehl, Antoine Studer, Mirjana Radosavljevic, Christine Kummerlen, Alexandra Monnier, Clotilde Boulay, Samira Fafi-Kremer, Vincent Castelain, Mickaël Ohana, Mathieu Anheim, Francis Schneider, Ferhat Meziani

**Affiliations:** 1 Hôpitaux Universitaires de Strasbourg, Service de Médecine Intensive-Réanimation, Nouvel Hôpital Civil, 1, place de l’Hôpital, F-67091 Strasbourg, Cedex France; 2 ImmunoRhumatologie Moléculaire, INSERM UMR_S1109, LabEx TRANSPLANTEX, Centre de Recherche d’Immunologie et d’Hématologie, Faculté de Médecine, Fédération Hospitalo-Universitaire (FHU) OMICARE, Fédération de Médecine Translationnelle de Strasbourg (FMTS), Université de Strasbourg (UNISTRA), Strasbourg, France; 3 Hôpitaux Universitaires de Strasbourg, Service d’imagerie 2, Hôpital de Hautepierre, Strasbourg, France; 4 Engineering Science, Computer Science and Imaging Laboratory (ICube), Integrative Multimodal Imaging in Healthcare, UMR 7357, University of Strasbourg-CNRS, Strasbourg, France; 5 INSERM (French National Institute of Health and Medical Research), UMR 1260, Regenerative Nanomedicine (RNM), FMTS, Strasbourg, France; 6 Hôpitaux Universitaires de Strasbourg, Service de Médecine Intensive-Réanimation, Hautepierre, Strasbourg, France; 7 Hôpitaux Universitaires de Strasbourg, Groupe Méthodes en Recherche Clinique (GMRC), Hôpital Civil, Strasbourg, France; 8 Laboratoire d’immunologie, Hôpitaux Universitaires de Strasbourg, Strasbourg, France; 9 Service de Neurologie, Hôpitaux Universitaires de Strasbourg, Strasbourg, France; 10 Institut de Génétique et de Biologie Moléculaire et Cellulaire (IGBMC), INSERM-U964/CNRS-UMR7104/Université de Strasbourg, Illkirch, France; 11 Fédération de Médecine Translationnelle de Strasbourg (FMTS), Université de Strasbourg, Strasbourg, France; 12 Hôpitaux Universitaires de Strasbourg, Laboratoire de Virologie Médicale, Strasbourg, France; 13 Radiology Department, Nouvel Hôpital Civil, Strasbourg University Hospital, Strasbourg, France

**Keywords:** COVID-19, Delirium, Encephalopathy, ICU, MRI

## Abstract

**Background:**

Neurotropism of SARS-CoV-2 and its neurological manifestations have now been confirmed. We aimed at describing delirium and neurological symptoms of COVID-19 in ICU patients.

**Methods:**

We conducted a bicentric cohort study in two French ICUs of Strasbourg University Hospital.

All the 150 patients referred for acute respiratory distress syndrome due to SARS-CoV-2 between March 3 and May 5, 2020, were included at their admission. Ten patients (6.7%) were excluded because they remained under neuromuscular blockers during their entire ICU stay. Neurological examination, including CAM-ICU, and cerebrospinal fluid analysis, electroencephalography, and magnetic resonance imaging (MRI) were performed in some of the patients with delirium and/or abnormal neurological examination. The primary endpoint was to describe the incidence of delirium and/or abnormal neurological examination. The secondary endpoints were to describe the characteristics of delirium, to compare the duration of invasive mechanical ventilation and ICU length of stay in patients with and without delirium and/or abnormal neurological symptoms.

**Results:**

The 140 patients were aged in median of 62 [IQR 52; 70] years old, with a median SAPSII of 49 [IQR 37; 64] points. Neurological examination was normal in 22 patients (15.7%). One hundred eighteen patients (84.3%) developed a delirium with a combination of acute attention, awareness, and cognition disturbances. Eighty-eight patients (69.3%) presented an unexpected state of agitation despite high infusion rates of sedative treatments and neuroleptics, and 89 (63.6%) patients had corticospinal tract signs. Brain MRI performed in 28 patients demonstrated enhancement of subarachnoid spaces in 17/28 patients (60.7%), intraparenchymal, predominantly white matter abnormalities in 8 patients, and perfusion abnormalities in 17/26 patients (65.4%). The 42 electroencephalograms mostly revealed unspecific abnormalities or diffuse, especially bifrontal, slow activity. Cerebrospinal fluid examination revealed inflammatory disturbances in 18/28 patients, including oligoclonal bands with mirror pattern and elevated IL-6. The CSF RT-PCR SARS-CoV-2 was positive in one patient. The delirium/neurological symptoms in COVID-19 patients were responsible for longer mechanical ventilation compared to the patients without delirium/neurological symptoms. Delirium/neurological symptoms could be secondary to systemic inflammatory reaction to SARS-CoV-2.

**Conclusions and relevance:**

Delirium/neurological symptoms in COVID-19 patients are a major issue in ICUs, especially in the context of insufficient human and material resources.

**Trial registration:**

NA.

## Introduction

Patients infected with severe acute respiratory syndrome coronavirus-2 (SARS-CoV-2), also known as coronavirus disease 2019 [COVID-19], mainly develop respiratory and digestive symptoms [1, 2]. However, due to similarity of viral structure and infection pathways [3], it has been suggested early in the epidemics that SARS-CoV-2 may also invade the central nervous system and be responsible for neurological signs [4], like other coronaviruses do [5–7]. Most β-coronaviruses, including SARS-CoV, have a neuroinvasive propensity [8]. Indeed, the SARS-CoV invades cells by using a cellular receptor angiotensin-converting enzyme 2 (ACE2), which may be expressed in ciliated upper respiratory cells and type II pneumocytes, responsible for the respiratory manifestations, but also in the central nervous system (neurons and glial cells), and endothelial cells [7, 9, 10]. Several data in humans and animals suggest that coronaviruses may have neurotropic effects and mainly affect brainstem and medullary cardiorespiratory center [10–12]. Coexisting brain lesions were also described in piglets infected with a transmissible gastroenteritis coronavirus, responsible for non-supportive encephalitis [11]. In a recent series of autopsies [13], von Weyhern et al. also described a pronounced central nervous system involvement with lymphocytic panencephalitis, meningitis, diffuse petechial hemorrhages, and brainstem neuronal cell damage. Furthermore, presence of SARS-CoV particles was finally demonstrated in the brain of patients with SARS [14].

In a descriptive study, Chen et al. reported the epidemiological and clinical characteristics of 99 cases of 2019 COVID-19 pneumonia in Wuhan, with headache in 8 patients (8%) and confusion in 9 patients (9%). Mao et al. [15] also retrospectively described neurological manifestations of SARS-CoV-2 infection in 78 of 214 (36.4%) patients with confirmed diagnosis of COVID-19 and hospitalized in Wuhan, China. The authors showed that 53 (24.8%) of the patients suffered from central nervous system symptoms, including dizziness, headache, and impaired consciousness. The authors also reported that neurologic symptoms were more common in patients with severe infection (45.5 vs. 30.0%, *p* = 0.02), with more cerebrovascular diseases and impaired consciousness. However, no analysis of cerebrospinal fluid, no brain magnetic resonance imaging, and only a few brain CT were performed. We have recently shown that most patients (84%) admitted to intensive care units for acute respiratory distress syndrome (ARDS) due to COVID-19 may develop neurological features, mainly delirious manifestations [16].

ICU delirium includes fluctuating disturbances in attention and cognition developing in a short period that are not explained by pre-existing neurocognitive disorder [17]. In survivors of critical illness, delirium has been shown to be associated with worse outcomes in critically ill patients with longer hospital stays, increased risk of long-term neurocognitive sequelae and neuropsychiatric disorders, and death [18].

In COVID-19, both the neurotropism of SARS-CoV-2 and its neurological manifestations have now been confirmed [4], although the mechanisms involved in these alterations are still debated. Based on the comprehensive clinical examination, analysis of cerebrospinal fluid (CSF), electroencephalography (EEG), and brain magnetic resonance imaging (MRI) of a homogeneous prospective cohort of patients admitted in ICU for ARDS due to SARS-CoV-2 infection, we aimed at describing the incidence of delirium AND/OR abnormal neurological examination and compare the outcome of these patients to patients without delirium or any neurological symptoms.

## Patients and methods

### Patients

Between March 3 and May 5, 2020, all the patients referred for ARDS [19] due to SARS-CoV-2 were prospectively included at admission in two ICUs from a French tertiary hospital in Strasbourg. Follow-up was performed until June 29, 2020. There was no exclusion criterion. Patients were managed following current guidelines [20], without specific therapeutic intervention.

The local ethics committee of Hospital University of Strasbourg approved the study (reference CE: 2020-35). In light of the clinical and epidemiological context, oral consent for the use of medical data could not be obtained for all patients, but was confirmed systematically by a relative and after the critical stage by the patient itself or its relative in case of death.

The demographic characteristics, medical history, and symptoms were reported. Clinical examination was performed daily by at least one senior experienced intensivist (experience ranging from 5 to 40 years). CSF analysis, EEG, and brain MRI were also studied (see Additional Fig. 1). The electroencephalographic and imaging data were reviewed by a trained team of two neurologists and two neuroradiologists, respectively.

### Outcomes

The primary endpoint was to describe the incidence of delirium AND/OR abnormal neurological examination, occurring at any time during their ICU stay in patients admitted to ICU for ARDS due to COVID-19.

The secondary endpoints were to describe the type delirium (hypoactive/hyperactive) [21] and to compare the duration of invasive mechanical ventilation (in days), ICU length of stay (in days), and mortality in patients with and without delirium AND/OR other neurological symptoms.

### Real-time reverse transcriptase PCR tests for COVID-19

Quantitative real-time reverse transcriptase PCR tests for COVID-19 nucleic acid were performed on nasopharyngeal swabs of all patients and in CSF in patients who had a lumbar puncture (ref: https://www.who.int/docs/default-source/coronaviruse/real-time-rt-pcr-assays-for-the-detection-of-sars-cov-2-institut-pasteur-paris.pdf?sfvrsn=3662fcb6_2).

### Richmond Agitation-Sedation Scale (RASS) and Confusion Assessment Method for the ICU (CAM-ICU)

Beginning at ICU admission, patients were assessed every 4 h by a nurse for level of consciousness using the Richmond Agitation-Sedation Scale [22]. All patients with a RASS between − 3 and + 4 were screened for confusion using the CAM-ICU twice a day by an intensivist [23]. CAM-ICU could not be performed in comatose patients (RASS − 4: unresponsive to voice but responsive to physical stimulation/RASS − 5: unresponsive to voice and physical stimulation).

Delirium was defined by a positive CAM-ICU at least once during ICU stay and was classified in hypo- or hyperactive delirium according to clinical presentation [21].

### EEG monitoring

Electroencephalography was performed in comatose patients who had unexplained and persistent altered consciousness after prolonged sedation discontinuation (> 3 days) to rule out nonconvulsive seizures [24] and in patients who underwent brain MRI and/or CSF examination.

### Magnetic resonance imaging

Brain MRI was performed in patients with the most severe and persistent delirium (> 3 days during ICU stay) and/or abnormal neurological examination. Brain MRI was only feasible if patients were hemodynamically stable (i.e., patients without catecholamines) and non-hypoxemic (FiO_2_ < 40% and PEEP < 8 or < 4 L/min oxygen) and without contra-indication to MRI. Patients underwent a gadolinium-enhanced brain MRI on a 3T MRI scanner (Achieva 3Tx, Philips, Best, The Netherlands, or SIGNA HDX 3T, GE, Milwaukee, USA). Acquisition parameters are summarized in Additional file 1.

### Analysis of cerebrospinal fluid

Analysis of CSF was performed in the same patients after brain MRI/CT, in the absence of contra-indication to lumbar puncture (e.g., therapeutic anticoagulation).

### Statistical analysis

Continuous variables are presented as median with the first and third quartile of the distribution and were compared using non parametric Wilcoxon tests. Categorical variables are described as numbers and proportions and were compared using Pearson’s *χ*^2^ tests or Fisher’s exact tests depending on theoretical numbers. Comparisons of the mechanical ventilation duration and ICU length of stay were performed using a multivariable gamma regression model. Goodness of fit for gamma distribution was assessed with histogram and QQ (quantile-quantile) plot. An adjustment was realized on the potentially confounding factors (age, sex, neurological medical history, SOFA, SAPS II, chronic renal diseases, and antiviral treatment). Results are presented as means ratios (MR) with their 95% confidence intervals. A *p* value < 0.05 was considered as statistically significant. As the findings should be interpreted as exploratory, the analyses have not been adjusted for multiple comparisons. All the analyses were performed using R software version 3.6.0. R Core Team (2019). R: A language and environment for statistical computing. R Foundation for Statistical Computing, Vienna, Austria. URL https://www.R-project.org/.

## Results

### Incidence of delirium and other neurological symptoms

**Fig. 1:**
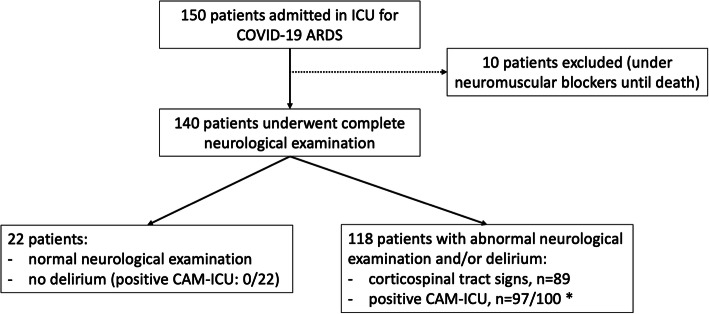
Flow chart. Asterisk indicates CAM-ICU was performed in 122/140 patients (87.1%). Four patients could not be evaluated because they did not speak French and 14 patients died without being scored (RASS − 4/− 5). CSF, cerebrospinal fluid; EEG, electroencephalogram; MRI, magnetic resonance imaging

One hundred and fifty consecutive patients admitted on ICU were included in the study (Fig. 1). Ten patients (6.7%) were excluded, because they could not be appropriately evaluated as they remained under neuromuscular blockers during their ICU stay, until death. ICU mortality rate was 15.6% (21/140 patients).

One hundred and eighteen patients (84.3%) displayed delirium (median RASS: 0.0 [0.0; 1.0]) and/or abnormal neurological exam at any time during ICU stay; 22 of them (18.6%) already displayed delirium and/or corticospinal tract signs at ICU admission. Table 1 compares baseline characteristics of these 118 patients with “delirium AND/OR abnormal neurological examination” to those of the 22 patients with “normal neurological examination AND no delirium” and Table 2 their outcome.

**Table 1:** Baseline characteristics of patients

	All patients (***n*** = 140)	No delirium and normal neurological examination (***N*** = 22)	Delirium and/or abnormal neurological examination (***N*** = 118)	***p***
**Age - median [IQR]**	62 [52; 70]	65 [48; 71]	62 [52; 71]	0.302
**Male (*n*, %)**	100 (71.4)	11 (50.0)	89 (75.4)	0.015
**Medical history**				
**Neurological medical history (*n*, %)**	22 (15.7)	4 (18.1)	18 (15.3)	0.707
Stroke/transient ischemic attack	9 (6.4)	0 (0.0)	9 (8.0)	0.354
Partial epilepsy	2 (1.4)	0 (0.0)	2 (1.8)	1
Mild cognitive alteration	4 (2.9)	1 (4.5)	3 (2.7)	0.499
Migraine	5 (3.6)	1 (4.5)	4 (3.4)	0.580
Trauma brain injury	2 (1.4)	1 (4.5)	1 (0.9)	0.291
Aneurysm	1 (0.7)	1 (4.5)	0 (0.0)	0.157
**Cardiovascular diseases—*n*(%)**	70 (50.0)	12 (54.5)	58 (49.2)	0.642
**Malignancies/hemopathies—*n*(%)**	21 (15.0)	5 (22.7)	16 (13.6)	0.423
**Immune diseases—*n*(%)**	4 (2.9)	2 (9,1)	2 (1.7)	0.233
**Diabetes—*n*(%)**	21 (15.0)	3 (13.6)	18 (15.3)	1
**Chronic liver disease—*n*(%)**	2 (1.4)	0 (0.0)	2 (1.7)	1
**Chronic renal disease—*n*(%)**	9 (6.4)	3 (13.6)	6 (5.1)	0.300
**Respiratory disease—*n*(%)**	22 (15.7)	2 (9,1)	20 (16.9)	0.566
Chronic obstructive pulmonary disease	2 (1.4)	0 (0.0)	2 (1.7)	1
Asthma	5 (3.6)	1 (4.5)	4 (3.4)	1
Obstructive sleep apnea	16 (11.4)	1 (4.5)	15 (12.7)	0.482
**SAPS II—median [IQR]**	49 [37; 64]	51 [34; 61]	49 [38; 63]	0.647
**SOFA—median [IQR]**	7 [4; 8]	6 [4; 8]	7 [5; 8]	0.486
**Antiviral treatments—*n*(%)**				
Lopinavir + ritonavir	46 (32.9)	5 (22.7)	41 (34.7)	0.271
Remdesivir	11 (7.9)	0 (0.0)	11 (9.3)	0.282
Hydroxychloroquine/azithromycine	52 (37.1)	8 (36.4)	44 (37.3)	0.934
Tocilizumab	3 (2.1)	1 (4.5)	2 (1.7)	0.807
Anakinra	1 (0.7)	0 (0.0)	1 (0.8)	1
Dexamethasone	1 (0.7)	0 (0.0)	1 (0.8)	1
None	25 (17.9)	7 (31.8)	18 (15.3)	0.130
**Positive SARS-CoV-2 RT-PCR in nasopharyngeal swabs**	140 (100)	22 (100)	118 (100)	1
**Chest CT scan suggestive of SARS-CoV-2 infection**	139 (99.3)	21 (95.5)	118 (100)	0.157

*ICU* intensive care unit, *SOFA* Sequential Organ Failure Assessment, *RT-PCR* real-time reverse transcriptase polymerase chain reaction, *SAPSII* simplified acute physiology score II

**Table 2:** Outcome of the patients

	All patients (***n*** = 140)	No delirium and normal neurological examination (***N*** = 22)	Delirium and/or abnormal neurological examination (***N*** = 118)	***p***
**Invasive mechanical ventilation**				
Duration (days)—median [IQR]	13 [9; 23]	9 [5; 17]	14 [10; 25]	0.011
Auto-extubation with immediate reintubation—*n* (%)	11 (7.9)	0 (0.0)	11 (9.3)	0.211
**ICU stay**				
ICU mortality—*n* (%)	21 (15.0)	2 (9.1)	19 (16.1)	0.634
Length of stay (days)—median [IQR]	15 [10; 25]	10 [6; 21]	15 [11; 25]	0.017
**Sedative treatments**				
Midazolam—*n* (%)	121 (86.4)	18 (81.8)	103 (87.3)	0.691
Midazolam (days)—median [IQR]	6 [3; 12]	4 [1; 9]	7 [4; 12]	0.095
Sufentanil—*n* (%)	138 (98.6)	20 (90.9)	118 (100)	0.047
Sufentanil—median [IQR]	10 [5; 15]	6 [1; 9]	11 [6; 16]	0.004
Propofol—*n* (%)	83 (59.3)	8 (36.4)	75 (63.6)	0.017
Propofol—median [IQR]	2 [0; 6]	0 [0; 3]	2 [0; 7]	0.027

CAM-ICU was performed in 122/140 patients (87.1%). Four patients could not be evaluated because they did not speak French and 14 patients died without being scored (RASS − 4/− 5). Delirium was diagnosed in 97/122 patients (79.5%) based on a positive CAM-ICU at least once during ICU stay.

Neurological examination was abnormal in 89/140 patients (63.6%), with corticospinal tract signs including diffuse enhanced, polykinetic tendon reflexes, ankle clonus, and bilateral extensor plantar reflexes. There was no meningeal syndrome, movement disorder, oculomotor abnormality, or fasciculation. These corticospinal tract signs persisted until ICU discharge in 75/140 patients (53.6%).

### Type of delirium in COVID-19 patients

On the 97/122 patients diagnosed with delirium based on a positive CAM-ICU, 84 patients (86.6%) had hyperactive delirium, while the others had hypoactive delirium.

Indeed, as soon as neuromuscular blockers were stopped, an unusual state of agitation (RASS + 3/+ 4) was assessed at least 1 day in 84 of the 122 patients assessed with CAM-ICU and in the 4 non-speaking patients (88 patients/126, 69.8%) in whom CAM-ICU could not be performed. This agitation required prolonged use of neuroleptic and sedative agents during a median of 5 [3; 10] days, preventing ventilator weaning and responsible for accidental extubation in 11/140 patients (7.9%).

### Prognosis of patients suffering from delirium and/or other neurological symptoms

The 11/11 patients experiencing auto-extubation required immediate reintubation because of an acute respiratory failure despite noninvasive ventilation and kinesitherapy. All these patients suffered from delirium on the day of auto-extubation. Auto-extubation/reintubation was followed by worsening of hypoxemia (delta PaO_2_/FiO_2_ before/after auto-extubation/reintubation: − 60), because of subsequent bad patient-ventilator synchrony requiring re-initiation of sedation in 9/11 patients.

Duration of invasive mechanical ventilation was significantly longer in patients with delirium and/or abnormal neurological examination compared to patients with normal neurological examination and without delirium (means ratio: 1.49 [1.01; 2.20], *p* = 0.045) and ICU length of stay tended to be longer (1.38 [0.95; 2.01], *p* = 0.092) (Table 2). Mortality rate difference between groups did not reach statistical significance (16.1 versus 9.1%) (Table 2).

### Electroencephalography revealed unspecific abnormalities

Forty-two EEG were performed in 42 out the 118 patients with delirium and/or abnormal neurological examination. Five EEG were normal. Twenty-six EEG revealed unspecific abnormalities with low voltage, rapid rhythm, and lack of asymmetry in good accordance with a context of confusion and sedation. The remaining 11 exams showed diffuse, especially bifrontal, slow activity. No patient suffered from convulsive status epilepticus.

### MRI showed enhancement of subarachnoid spaces, intraparenchymal abnormalities, and perfusion abnormalities

Brain MRI was performed in 32 out of the 118 patients. Four MRI were excluded because not interpretable due to artifacts on movements. Among these 32 patients, six had neurological disease in their medical history prior to ICU admission: 2 patients had stroke (and had no previous MRI), 2 had transient ischemic attack with normal previous imaging, and 2 patients had partial epilepsy. However, we have not described any abnormalities related to these pre-existent neurological diseases.

**Fig. 2:**
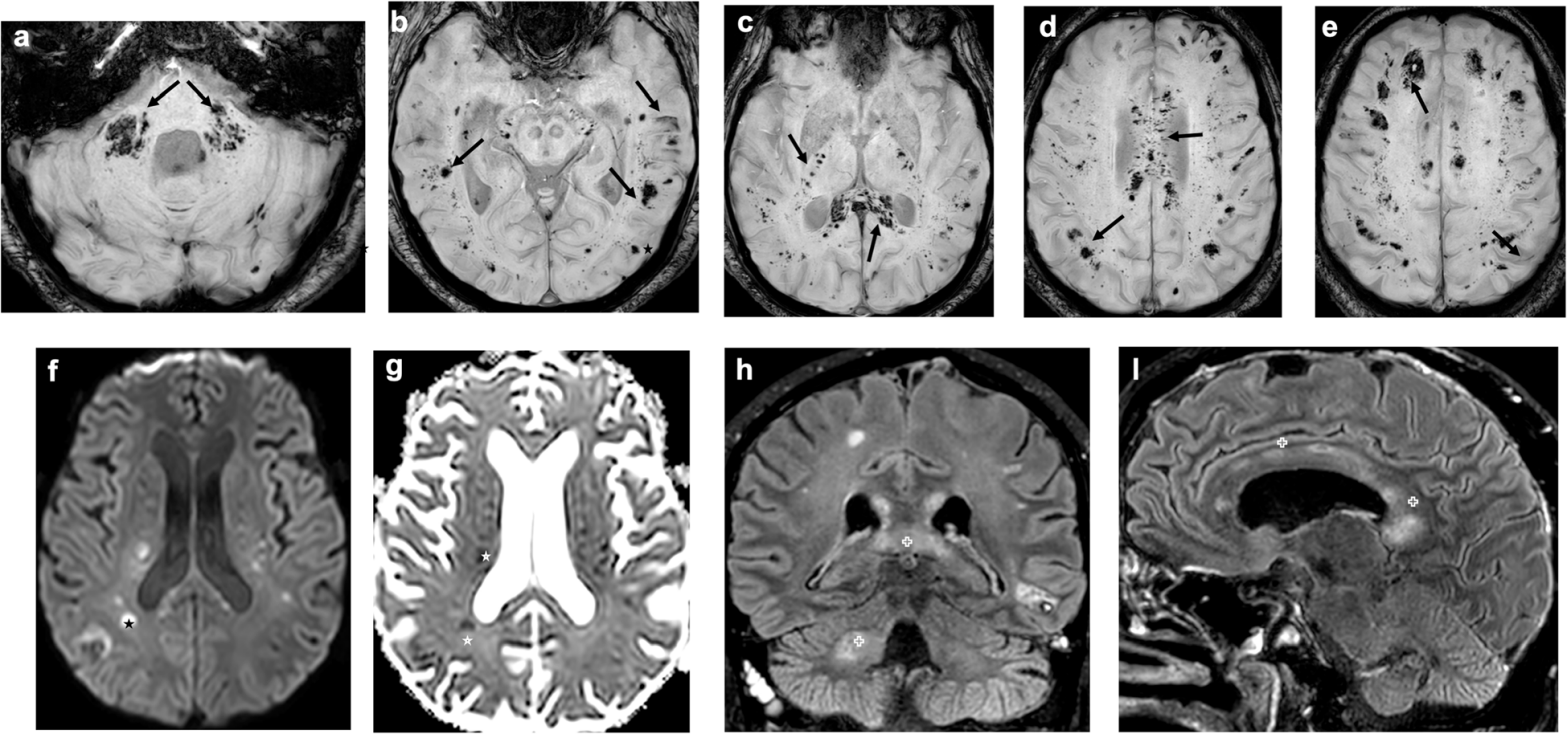
Axial SWI (**a**–**e**), axial diffusion (**f**), apparent diffusion coefficient (ADC) (**g**), coronal (**h**), and sagittal (**i**) FLAIR-weighted MR images: multiple infra and supratentorial white matter microhemorrhages (arrows), associated with FLAIR (cross) and diffusion (star) hyperintensities

**Fig. 3:**
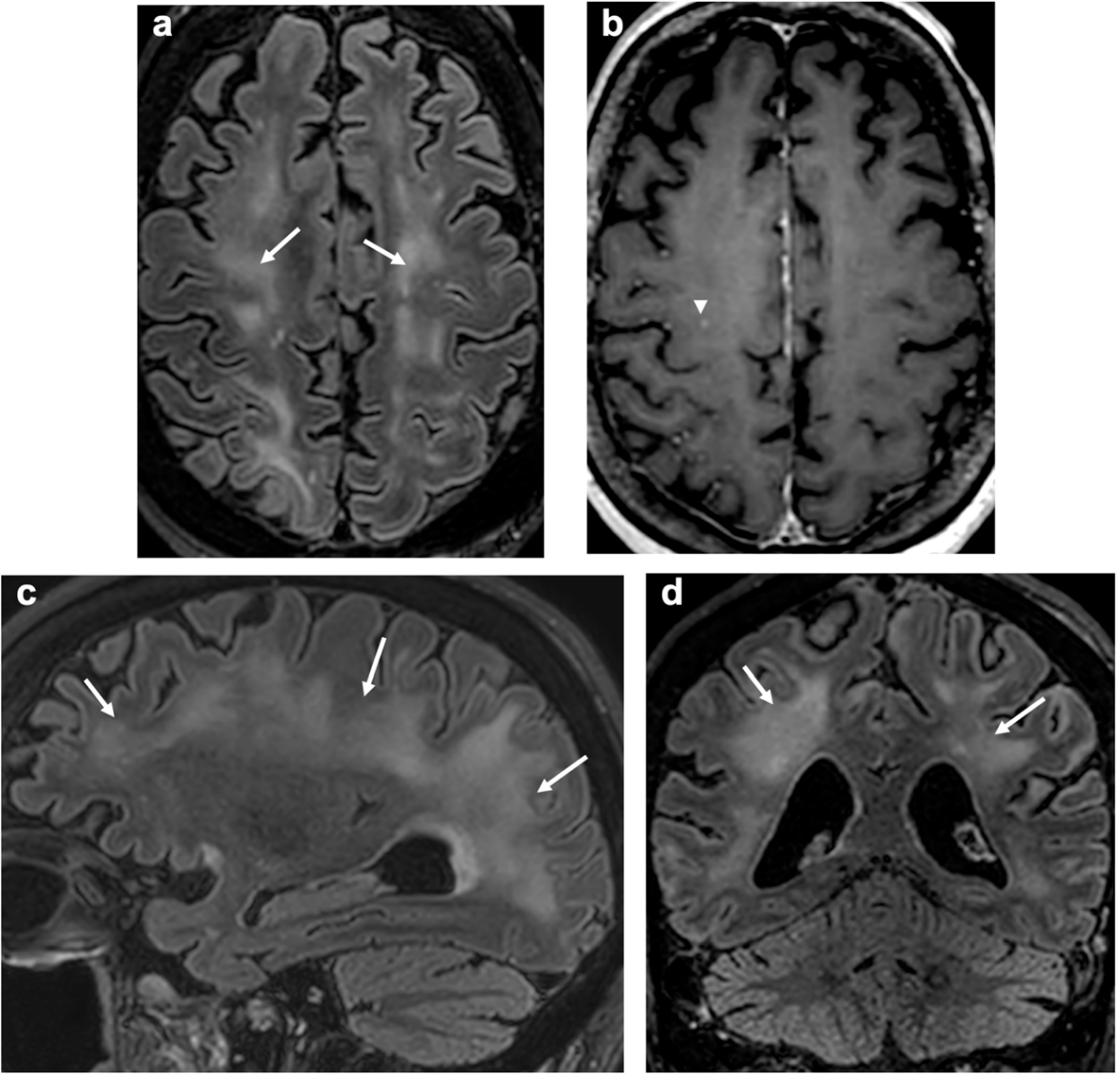
Axial, sagittal, and coronal FLAIR (**a**, **c**, **d**) and axial post-contrast T1 (**b**)-weighted MR images: extensive white matter confluent FLAIR hyperintensities (arrow), with small foci of contrast enhancement (arrow head)

Among the 28 MRIs, eight patients presented intraparenchymal, predominantly white matter abnormalities: 7 had white matter microhemorrhages (Fig. 2), associated to left frontal intraparenchymal hematoma in 1 case. Four patients had FLAIR hyperintensities, with small foci of contrast enhancement in 2 cases (Fig. 3) and diffusion hyperintensities in 2 cases.

Seventeen patients (60.7%) presented with subarachnoid spaces FLAIR and T1 contrast enhancement, hyperintensity that were not present on precontrast T1 or FLAIR images.

Three patients had a cerebral ischemic stroke, which was acute in two cases with hyperintensity on diffusion-weighted imaging and decreased diffusion coefficient and probably preexisting to COVID-19 infection in the other case because of ADC increase, lack of contrast enhancement, and absence of mass effect.

Twenty-six patients underwent perfusion MRI (Arterial Spin Labeling-ASL). Cerebral blood flow (CBF) maps demonstrated abnormal CBF pattern in 17/26 patients (65.4%) (see Additional Fig. 2).

### Cerebrospinal fluid analysis revealed inflammatory disturbances in two thirds of the patients

A lumbar puncture was performed in 25 out of the 32 patients who underwent MRI (Table 3). CSF aspect was transparent, colorless, and analysis was unremarkable for glucose, proteins, and lactate in 19 patients. Cytology was normal (no leukocytes, < 5 erythrocytes) in all patients. Identical oligoclonal bands in the CSF and in the serum consistent with mirror pattern were described in 13 patients. Nine patients had elevated intrathecal IgG levels, with normal blood IgG levels and mildly elevated protein levels. The RT-PCR SARS-CoV-2 was negative in cerebrospinal fluid, except in one patient who had negative RT-PCR SARS-CoV-2 in blood (excluding a breach). Bacterial cultures were sterile and viral research (HSV-1, HSV2, enterovirus) were also negative in all patients. Interleukins 6 and 10 were measured in CSF (Table 3).

**Table 3:** Cerebrospinal fluid analysis

	All patients (***n*** = 25)
**CSF analysis—median [IQR]**	
Nucleated cell count (cells/mm^3^)**—***normal range < 5*	1 [0; 2]
CSF protein level (g/L)**—***normal range 0.15–0.45 g/L*	0.33 [0.26; 0.59]
CSF glucose level (g/L)	0.89 [0.75; 1.28]
CSF lactate level (mmol/L)**—***normal range 1.2–2.1 mmol/L*	1.29 [1.09; 1.80]
CSF IgG level (mg/L)**—***normal range 10–34 mg/L*	32.3 [19.2; 50.3]
CSF albumin level (mg/L)**—***normal range 130–350 mg/L*	184 [121; 308]
Albumin ratio CSF/serum X 10^3^**—***normal range < 8.5*	7.5 [5.8; 11.6]
CSF Interleukin-6 level (pg/mL)**—***normal range < 13 pg/mL*	8.9 [2.7; 13.5]
CSF Interleukin-10 level (pg/mL)**—***normal range < 3 pg/mL*	0.0 [0.0; 0.1]
CSF Interferon gamma (pg/mL)**—***normal range < 80 pg/mL*	0.6 [0.4; 0.7]
**CSF abnormalities—number of patients (%)**	
Abnormal CSF analysis	18 (72.0)
Elevated nucleated cell count	3 (12.0)
Elevated CSF protein levels	8 (32.0)
Elevated CSF albumin level	5 (20.0)
Elevated albumin ratio CSF/serum	4 (16.0)
Elevated CSF IgG	9 (36.0)
Oligoclonal bands with mirror pattern	13 (52.0)
Elevated interleukin-6 level	7 (28.0)
Elevated interleukin-10 level	2 (8.0)
Elevated interferon gamma level	0 (0.0)
Positive SARS-CoV-2 RT-PCR in CSF	1 (4.0)

*CSF* cerebrospinal fluid, *RT-PCR* real-time reverse transcriptase polymerase chain reaction

## Discussion

Herein, we describe the high frequency of delirium and/or neurological symptoms (84.3%) in patients admitted to ICU for ARDS due to COVID-19 and their worse prognosis compared to patients without delirium and with normal neurological examination. Indeed, delirium and/or neurological symptoms led to sustained invasive mechanical ventilation and unusually high doses of sedations and neuroleptics, whereas patients without delirium and with normal neurological examination could be extubated and discharged earlier from ICU. The delirium also increased the risk of life-threatening accidental extubation and was associated to worsening of hypoxemia after reintubation, thus maybe contributing to increase the duration of the ventilator weaning process. Mortality rate difference between groups did not reach statistical significance (16.1versus 9.1%), although it may be due to the small number of patients in our cohort.

Because COVID-19 mostly affects men [25–27], possibly because of a large number of ACE2-expressing cells in their lung (report not peer-reviewed) [28], most ICU patients with COVID-19 delirium were men (Table 1).

Whether the acquired acute global disturbance in cognition we describe should be referred to only as delirium or even as encephalopathy may be questioned. Delirium is mainly characterized by acute attention, awareness, and cognition disturbances, while acute encephalopathy, that may not be diagnosed at bedside, is used “to describe a rapidly developing (in less than 4 weeks) pathobiological brain process which is expressed clinically as either subsyndromal delirium, delirium or coma and may have additional features, such as seizures or extrapyramidal signs” [17]. Beyond the neurological signs we have described, the clinical diagnosis of COVID-19 encephalopathy is strengthened by paraclinical features that allowed to delineate a pathobiological process: (i) MRI findings revealing perfusion abnormalities, (ii) electroencephalographic abnormalities, (iii) hints for inflammatory process in CSF, and (iv) the lack of other identified cause of delirium beyond positive SARS-CoV2 RT-PCR in all patients. Some of our patients have therefore developed an acute COVID-19 encephalopathy. We can however not affirm that all the patients with neurological symptoms fulfilled the diagnostic criteria for encephalopathy in our cohort, considering that only part of them have been submitted to paraclinical exams.

Perfusion abnormalities have been previously described in ICU and non-ICU patients with COVID-19, describing hypoperfused areas, predominantly in the temporal lobes and to a lesser extent in the frontal lobes [16, 29].

Delirium (positive CAM-ICU) or acute encephalopathy, as defined above, may relate to systemic inflammatory reaction to SARS-CoV-2 rather than to SARS-CoV-2 itself. Knowing that we have not analyzed the CSF of all patients in our cohort, the hypothesis of an inflammatory component in the emergence of the COVID-19 delirium is supported by (i) the absence of RNA viral load in CSF at the time of the diagnosis of COVID-19 except in one patient; (ii) CSF analysis revealing mirror pattern of oligoclonal bands, elevated protein and IgG level, and elevated proinflammatory cytokine IL-6; and (iii) brain imaging showing subarachnoid contrast enhancement suggestive of abnormal permeability of the blood meningeal barrier associated with the encephalopathy. One may speculate that a systemic immune event could be responsible for such abnormal permeability in the same way that the cytokine storm is involved in the occurrence of the SARS-CoV-2 infection [1].

The high frequency and reproducibility of neurological signs in our patients reinforces the hypothesis that COVID-19 may be responsible at least for delirium and in some cases for encephalopathy. Furthermore, other causes of delirium/encephalopathy were excluded (for instance iatrogenic, alcoholic, or metabolic). There was no stroke except in 3 patients that could not explain the clinical signs. In the same way, the patients with history of epilepsy had not ictal abnormalities on electroencephalography. Iatrogenic cause was also excluded, because delirium and/or corticospinal tract signs were present in 22 (15.7%) patients on ICU admission, before administration of any treatment, and because such clinical findings are very unusual in ICU patients.

Based on CAM-ICU score, Salluh et al. [30] reported a prevalence of delirium of 32.3% in a multicenter study including 497 patients, while Khan et al. [31] more recently showed that delirium occurred in 16.5% out of the 2742 ICU patient included. Prevalence of ICU delirium is however highly variable among studies, depending of the definition and screening tools used, but also on the population studied. It was therefore suggested to reach up to 73% of the 48 patients with ARDS [32]. We have reported a very high prevalence (79.5%) of delirium in COVID-19 patients. Yet, the incidence of delirium in COVID-19 patients is still probably largely underestimated [33]. Indeed, most patients were intubated before ICU admission and already under neuromuscular blockers and delirium was diagnosed following an attempt of weaning of sedation when patients were recovering from respiratory failure.

Our results raised the hypothesis of an inflammatory component in the emergence of the COVID-19 delirium and/or neurological symptoms. Consistent with Mao et al. [15], our cohort also supports the hypothesis that severe patients were more likely to develop neurological symptoms. Li et al. [12] further suggested that the neuroinvasive potential of SARS-CoV2 may play a role in the respiratory failure of COVID-19 patients, which may explain the high prevalence of delirium in our patients who have been admitted on ICU for ARDS. Furthermore, in our cohort, there were hints for SARS-CoV2 encephalitis in 8 patients, with elevated CSF protein level, brain lesion on MRI and because CSF SARS-CoV2 RT-PCR was positive in one patient investigated.

Herein, laboratory investigations, electrophysiology, and especially brain imaging were helpful to understand the clinical findings. Seventeen patients presented with subarachnoid FLAIR and T1 contrast enhancement that were focal or diffuse. Alterations of the blood-brain barrier resulting from endothelial invasion [10] may explain the inflammatory findings in CSF analysis, as well as the negative RT-PCR in CSF. Some patients also presented cerebral, predominantly white matter abnormalities which were hemorrhagic in 7 patients. Our results are consistent with recently published data who demonstrated brain MRI abnormalities [34–37].

The main limitation of the study is the lack of comprehensive paraclinical tests in many patients and not all patients had the same set of studies performed. Yet, we could not perform more exams, because these were (i) extremely time-consuming (brain MRI) or not available (EEG/brain MRI), (ii) impossible in many patients because of hemodynamic/respiratory instability, agitation state, (iii) or contra-indicated (e.g., anticoagulant treatment and lumbar puncture). Further larger studies are thus needed to confirm the clinical and paraclinical characteristics of the COVID-19 delirium/encephalopathy, which could greatly participate to the burden caused by the unexpected SARS-CoV2 pandemic, and to confirm the generalization of our results, our cohort coming from one country population. In the same way, the identification of patients at risk of developing delirium as well as the underlying pathophysiological mechanisms should be also elucidated. Finally, we were not sure how to classify the aforementioned symptoms, whether delirium or encephalopathy.

The main strength of our study is to highlight the urgent need for post-ICU care reorganization. Indeed, in a context of severe COVID-19 pandemic overwhelming the capacities of ICUs [2, 38], one should bear in mind that COVID-19-associated delirium may lead to unusually long ICU stay and that it is not only a consequence of CoV2-induced ARDS [39]. Furthermore, whether patients will completely recover from these neurological symptoms is uncertain and should lead to organization of appropriate post-ICU care, including respiratory and neurological rehabilitation, and long-term medical follow-up.

## Conclusion

SARS-CoV2 infection may be frequently associated with COVID-19 delirium and/or neurological symptoms, leading to sustained sedation and mechanical ventilation thus markedly worsening the prognosis. Our study highlights the importance of the organization of adequate post-ICU care, including respiratory and neurological rehabilitation, and long-term medical follow-up, considering the high incidence of COVID-19 delirium and/or neurological symptoms, the risk of long-term neurocognitive sequelae, and neuropsychiatric disorders in survivors. Delirium and/or neurological symptoms may be due to systemic inflammatory reaction to SARS-CoV-2, although the pathophysiological mechanisms involved require further investigations.

## Supplementary information

**Additional file 1.** Supplemental information

## Data Availability

All data generated or analyzed during this study are included in this published article.

## Abbreviations

ACE2: Angiotensin-converting enzyme 2
ADC: Apparent diffusion coefficient
ARDS: Acute respiratory distress syndrome
CAM-ICU: Confusion Assessment Method for the ICU
CBF: Cerebral blood flow
COVID-19: Coronavirus disease 2019
CSF: Cerebrospinal fluid
DPP4: Dipeptidyl peptidase 4
DSM-5: Diagnostic and Statistical Manual of Mental Disorders
EEG: Electroencephalography
ICU: Intensive care unit
MR: Means ratios
MRI: Magnetic resonance imaging
QQ: Quantile-quantile
SAPSII: Simplified acute physiology score II
SARS-CoV-2: Severe acute respiratory syndrome coronavirus-2
SOFA: Sequential Organ Failure Assessment
RASS: Richmond Agitation-Sedation Scale
RT-PCR: Real-time reverse transcriptase PCR
