# Supplementary material for: Delirium and encephalopathy in severe COVID-19: a cohort analysis of ICU patients
Source: Crit Care. 2020 Aug 8;24:491. doi: 10.1186/s13054-020-03200-1 (PMC7414289; doi:10.1186/s13054-020-03200-1)
Supplement: Supplementary file 1 — Additional file 1. Supplemental information [file 13054_2020_3200_MOESM1_ESM.zip › Additional file 1.doc.pdf]

Additional file 1:

Supplemental methods:

Magnetic resonance imaging

Achieva 3Tx, Philips, Best, The Netherlands: All brain examinations were conducted using a 32 channel headcoil, with the following sequences:

- Sagittal T1 3D GE (TE=4.1ms, TR=8.8ms, flip angle=8°, TI 1000ms, slice thickness 0.85mm)
- Axial PD-T2 (TE=24.8ms (PD) – 120ms (T2), TR=6321ms, flip angle=90°, slice thickness 3mm)
- Axial Diffusion Weighted Imaging (TE = 92 ms, TR = 6912ms, flip angle = 90°, b = 1000, slice thickness= 3 mm, DTI with 6 gradient directions)
- Axial SWI (TE=7.2ms, TR=30ms, flip angle=17°, slice thickness 3mm)
- 3D TOF MR angiography (TE=5.8ms, TR=25ms, flip angle=20°, slice thickness 1.2mm)
- Pre gadolinium sagittal 3D FLAIR (TE=290ms, TR=4800ms, flip angle=90°, TI 1650ms, slice thickness 0.95mm) – performed in 3 out of 5 patients
- Arterial spin labelling perfusion MRI 3D PC ASL (TE=11ms, TR=4235ms, flip angle=90, slice thickness 6mm) – performed in 2 out of 5 patients

Intravenous injection of 0,1 mmol/Kg Gd-DOTA (Guerbet Laboratory, Aulnay sous bois, France) was performed, followed by

- dynamic susceptibility-weighted contrast-enhanced magnetic resonance perfusion imaging (DSC-MRI perfusion (SG, WC) (TE=8ms, TR=17ms, flip angle = 7°, slice thickness 3.6mm) – performed in 3 out of 5 patients
- Post gadolinium sagittal 3D FLAIR (TE=290ms, TR=4800ms, flip angle=90°, TI 1650ms, slice thickness 0.95mm)
- Post gadolinium sagittal 3D T1 GE (TE=4.1ms, TR=8.8ms, flip angle=8°, TI 1000ms, slice thickness 0.85mm) or 3D T1 SE (TE=70ms (34ms effect.), TR=370ms, flip angle=90°, slice thickness 1mm)

SIGNA HDX 3T, GE, Milwaukee, USA: All brain examinations were conducted using a 8 channel headcoil, with the following sequences:

- Sagittal T1 3D SE (TE=10,3ms, TR=550ms, slice thickness 1,2mm)

- Axial Diffusion Weighted Imaging (TE = 88.8 ms, TR = 11000ms, b = 1000, slice thickness= 4 mm, DTI with 6 gradient directions)
- Axial SWAN (TE=22,8ms, TR=38.6ms, flip angle=15°, slice thickness 2mm)
- 3D TOF MR angiography (TE=2.8ms, TR=24ms, flip angle=15°, slice thickness 1mm)
- Pre gadolinium sagittal 3D FLAIR (TE=122ms, TR=6000ms, slice thickness 1,2mm)
- Arterial spin labelling perfusion MRI 3D PC ASL (TE=9,8ms, TR=4599ms, TI=1525ms, slice thickness 4mm)

Intravenous injection of 0,1 mmol/Kg Gd-DOTA (Guerbet Laboratory, Aulnay sous bois, France) was performed, followed by

- dynamic susceptibility-weighted contrast-enhanced magnetic resonance perfusion imaging (DSC-MRI perfusion (TE=65ms, TR=2200ms, flip angle = 90°, slice thickness 4mm)
- Post gadolinium sagittal 3D FLAIR (TE=122ms, TR=6000ms, slice thickness 1,2mm)
- Post gadolinium sagittal 3D T1 SE (TE=10,3ms, TR=550ms, slice thickness 1,2mm)

Additional figure legends:

Additional Figure 1: Distribution of paraclinical exams and reasons for not doing each exam. MRI: magnetic resonance imaging, CSF: cerebrospinal fluid analysis

Additional Figure 2: ASL perfusion Cerebral Blood Flow (CBF) maps (a,b): Right and left internal temporal lobe hypoperfusion (arrows) (a), associated to right frontal lobe hypoperfusion (arrow) as compared to left frontal lobe (cross) (b).
