# Supplementary figures and images for: Delirium and encephalopathy in severe COVID-19: a cohort analysis of ICU patients
Source: Crit Care. 2020 Aug 8;24:491. doi: 10.1186/s13054-020-03200-1 (PMC7414289; doi:10.1186/s13054-020-03200-1)

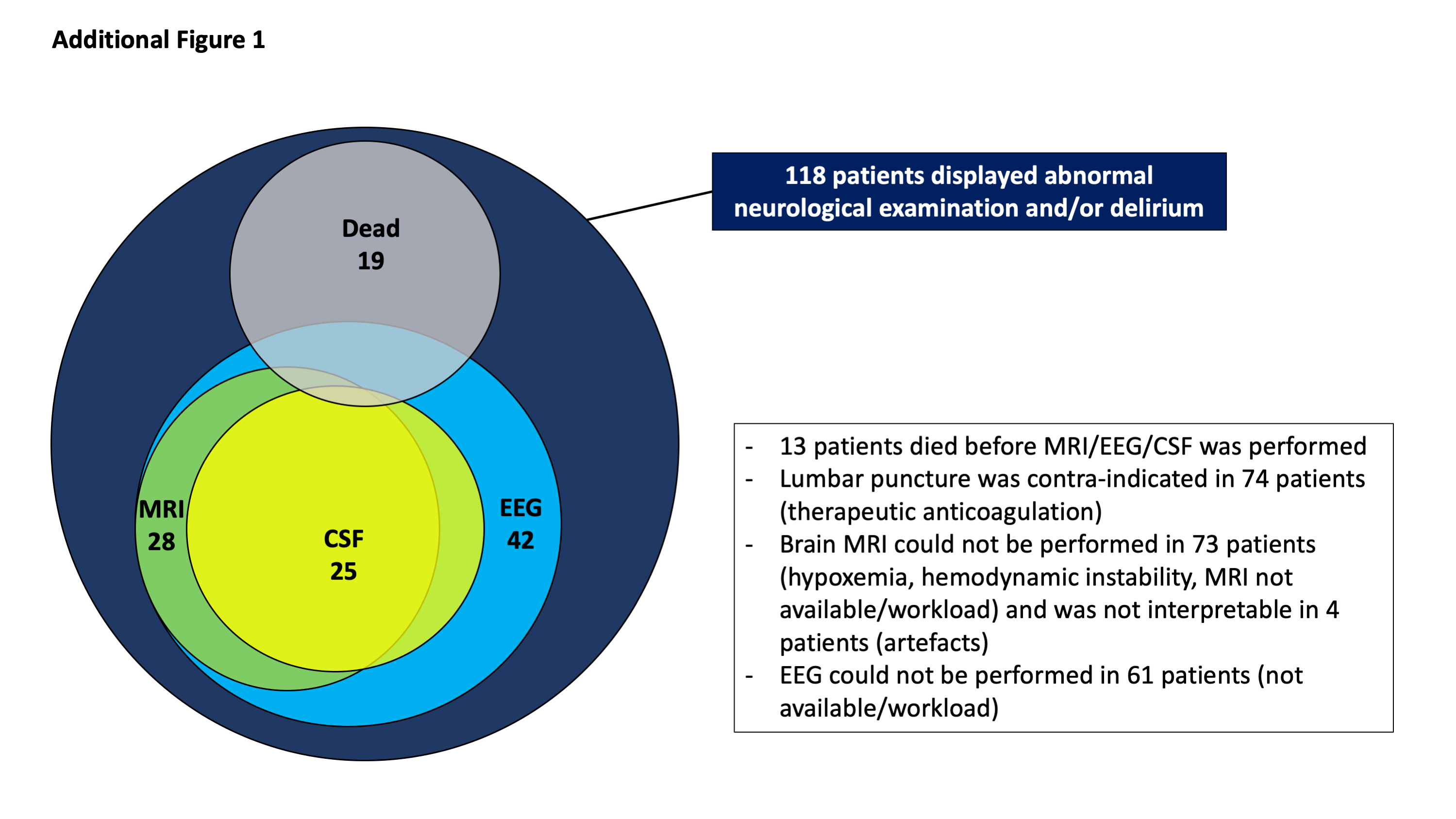

Supplement: Supplementary file 1 — Additional file 1. Supplemental information [file 13054_2020_3200_MOESM1_ESM.zip › Additional fig 1.tiff]

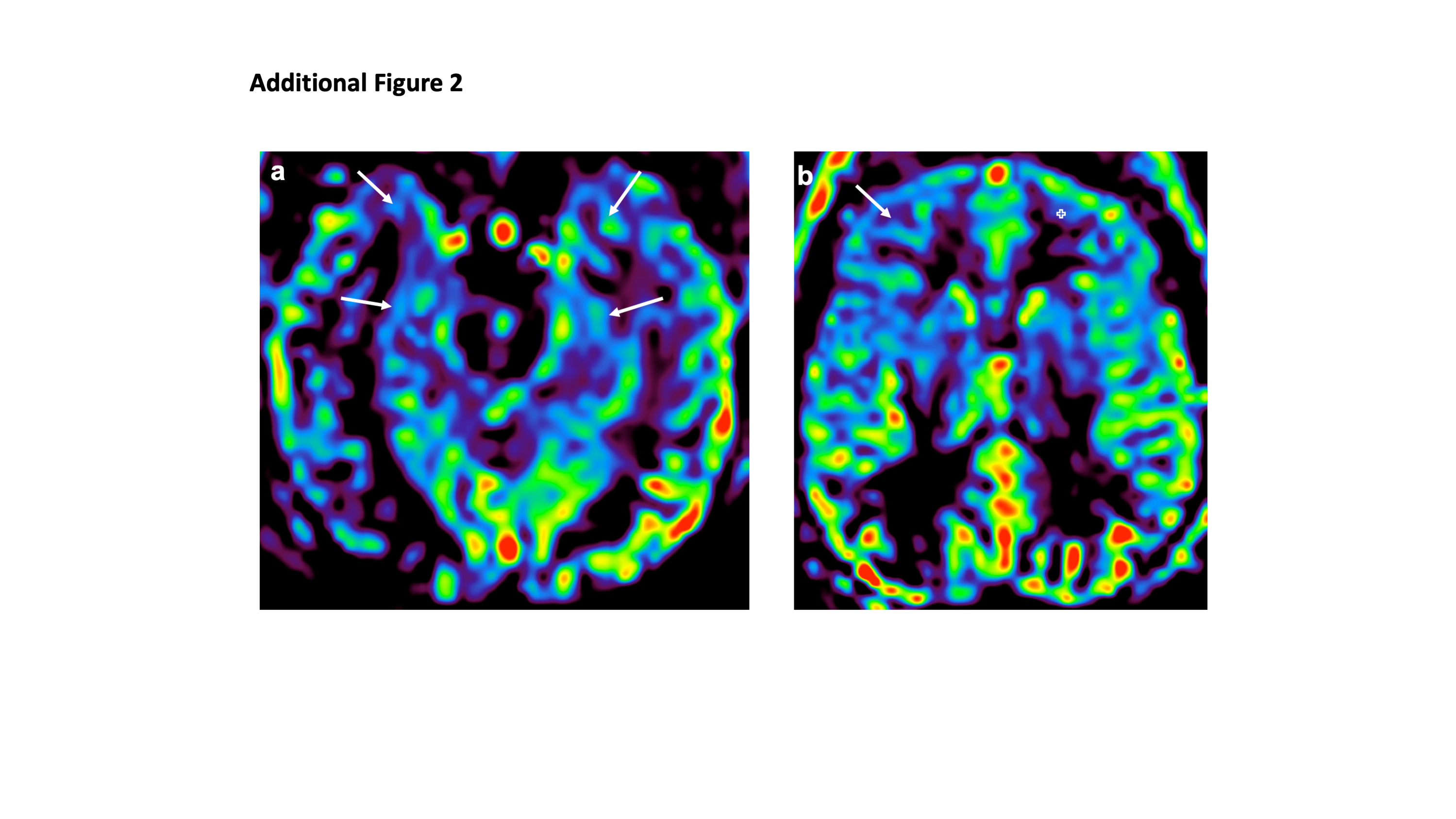

Supplement: Supplementary file 1 — Additional file 1. Supplemental information [file 13054_2020_3200_MOESM1_ESM.zip › additional figure 2.tiff]
